# Treating since the beginning?

**DOI:** 10.1186/1742-4690-9-S1-I15

**Published:** 2012-05-25

**Authors:** Jean-Pierre Routy

**Affiliations:** 1 McGill University, Montreal, Canada

## 

Like a pendulum, the therapeutic recommendations to initiate antiretroviral therapy (ART) have changed over the last two decades. Indeed, initiation of ART has swung from an initial “treat hard and early”, to a CD4 T cell count spanning from 200 to 500 and recently to a “as soon as the patient is ready”. Changes on the optimal timing to initiate ART were based on the following 1) Availability of new ART with increased potency, improved tolerance and reduced pill burden; 2) Evidence that controlled viral replication on ART is associated with a dramatic decrease in HIV transmission; 3) New insights on HIV pathogenesis and on the establishment and maintenance of HIV reservoirs.

Recent findings on HIV pathogenesis concerning the relationships between CD4 T cell counts, immune activation, non-infectious clinical events and cancers will be presented. Specifically, the influence of CD4 nadir on the quality of long-term immune reconstitution and viral reservoir persistence following ART initiation will be highlighted. We will also focus on the clinical relevance of maintaining certain CD4 T cell subsets like central memory pool for an optimal cytotoxic HIV-specific and vaccination responses. We will also revisit the tissue and cellular localizations of HIV reservoirs according to the time of treatment initiation. Furthermore, ethical considerations on a “patient-centered medicine” for early ART initiation will be discussed. Particularly, the advantages and inconveniences of a life-long treatment on a patient’s quality of life, drug-resistance development, long-term drug toxicity, cost and observance issues will be presented. The very early ART initiation reveals a new frontier 1) Allowing for some patients an ART-free viral control post-drug discontinuation era 2) Selecting optimal patients harboring a reduced HIV reservoir to be invited to participate in clinical trials aiming to HIV eradication. We are entering in a new era where “Treating early to be able to stop early” will be the focus of our future collaborating research efforts to vividly improve the life of HIV-infected people.

